# Lytic Release of Cellular ATP: Physiological Relevance and Therapeutic Applications

**DOI:** 10.3390/life11070700

**Published:** 2021-07-16

**Authors:** Ryszard Grygorczyk, Francis Boudreault, Olga Ponomarchuk, Ju Jing Tan, Kishio Furuya, Joseph Goldgewicht, Falonne Démèze Kenfack, François Yu

**Affiliations:** 1 Centre de Recherche du Centre Hospitalier de l’Université de Montréal (CRCHUM), Montréal, QC H2X 0A9, Canada; francis.boudreault.crchum@gmail.com (F.B.); ponomarchuk.olga.olegovna@gmail.com (O.P.); jj.tan@umontreal.ca (J.J.T.); joseph.goldgewicht@umontreal.ca (J.G.); falonne.demeze.kenfack@umontreal.ca (F.D.K.); 2 Département de Médecine, Université de Montréal, Montréal, QC H2X 0A9, Canada; 3 Graduate School of Medicine, Nagoya University, Nagoya 464-8601, Japan; furuya@med.nagoya-u.ac.jp; 4 Département de Radiologie, Radio-Oncologie et Médecine Nucléaire, Université de Montréal, Montréal, QC H2X 0A9, Canada; 5 Institut de Génie Biomédical, Université de Montréal, Montréal, QC H2X 0A9, Canada

**Keywords:** ATP release, purinergic signalling, cell lysis, hemolysis, membrane fragility, microbubble cavitation, sonoporation, ultrasound-targeted therapy

## Abstract

The lytic release of ATP due to cell and tissue injury constitutes an important source of extracellular nucleotides and may have physiological and pathophysiological roles by triggering purinergic signalling pathways. In the lungs, extracellular ATP can have protective effects by stimulating surfactant and mucus secretion. However, excessive extracellular ATP levels, such as observed in ventilator-induced lung injury, act as a danger-associated signal that activates NLRP3 inflammasome contributing to lung damage. Here, we discuss examples of lytic release that we have identified in our studies using real-time luciferin-luciferase luminescence imaging of extracellular ATP. In alveolar A549 cells, hypotonic shock-induced ATP release shows rapid lytic and slow-rising non-lytic components. Lytic release originates from the lysis of single fragile cells that could be seen as distinct spikes of ATP-dependent luminescence, but under physiological conditions, its contribution is minimal <1% of total release. By contrast, ATP release from red blood cells results primarily from hemolysis, a physiological mechanism contributing to the regulation of local blood flow in response to tissue hypoxia, mechanical stimulation and temperature changes. Lytic release of cellular ATP may have therapeutic applications, as exemplified by the use of ultrasound and microbubble-stimulated release for enhancing cancer immunotherapy in vivo.

## 1. Introduction

The release of cellular ATP and other purine and pyrimidine compounds, such as ADP, the nucleoside adenosine (Ado), UTP, UDP, and UDP-glucose, initiates the purinergic signalling pathway, which is an evolutionary conserved intercellular signalling system present in all living organisms. ATP regulates a vast range of physiological and pathophysiological processes by activating purinergic receptors on target cells, which include ionotropic (P2X) and G protein-coupled (P2Y) receptors. Ado exclusively activates G protein-coupled receptors of the P1 family (A_1_, A_2A_, A_2B_, and A_3_). While nucleotides act as primary messengers in intercellular communication, their actions are greatly expanded by stimulating the release of other extracellular messenger substances, such as neurotransmitters, hormones, cytokines, lipid mediators, nitric oxide, and reactive oxygen species [1].

The nucleotide release mechanisms are still debated. ATP, which plays a role as a stress-responsive signalling molecule, is liberated from cells in response to a variety of physical stimuli, especially mechanical stresses such as cell stretch and deformation, shear stress, and hypoosmotic swelling [2,3]. The release mechanisms may include non-lytic cell-regulated processes and lytic release due to membrane injury and cell lysis. Among cell-regulated processes, Ca^2+^-dependent exocytosis of ATP-containing vesicles/granules constitutes the most prominent non-conductive ATP release pathway in excitable and non-excitable cells [3,4]. However, currently, the conductive release mechanism involving a variety of putative ATP-conducting channels, including Pannexin-1, connexins, volume-regulated anion channel (VRAC), and calcium homeostasis modulator (CALHM) channels, attracts the most attention [5,6,7,8,9]. Historically this research area was plagued by several false-positive observations, as some early claims of conductive ATP release through the cystic fibrosis transmembrane conductance regulator (CFTR) channel or Gd^3+^-sensitive stretch-activated channels were not supported by later studies [10,11]. ATP-conducting properties have been functionally established for the voltage-dependent anion channel (VDAC) of the outer mitochondrial membrane by recording single-channel ATP-mediated currents [12], and much supportive evidence also exists for a porin-like maxi-Cl¯-channels [13,14]. It remains contentious, however, for several other channel candidates where definite evidence based on patch-clamp electrophysiology is still missing [13,15]. These suppositions may, at least in part, arise from the often underestimated or neglected “conductive component” of ATP release due to cell membrane damage and cell lysis. In the early days of purinergic signalling, before cell-regulated mechanisms were recognized, the lytic release of ATP was presumed to be the only mechanism of the release. Paradoxically, today it remains overlooked or not fully accounted for in the majority of studies.

In this review, we present examples of well-documented lytic ATP release that involves membrane rupture and cell lysis. In Section 2, we present evidence for a small lytic component of hypotonic shock-induced ATP release in alveolar A549 cells. Its contribution arises from the presence of fragile cells, especially after serum deprivation. In red blood cells (RBCs), ATP release appears entirely attributable to hemolysis regardless of the type of stimuli and will be discussed in Section 3. Lytic ATP release may also have therapeutic roles by contributing to purinergic signalling-dependent treatments. As an example, in Section 4, we describe the use of ultrasound and microbubble-induced ATP release for enhancing cancer immunotherapy.

## 2. Quantitative Evidence for Hypo-Induced Lytic ATP Release in Serum-Deprived A549 Cells

Human lung epithelial A549 cells are osmotically resilient and slowly secrete ATP during swelling without experiencing lysis, as documented in our recent study using a real-time imaging assay [16]. On the other hand, A549 cells rendered fragile by serum deprivation (0.1% FBS) become prone to membrane rupture under hypo-osmotic stress. In ATP imaging experiments, this was seen as striking punctual bursts of ATP secretion strongly co-localizing with the apparition of propidium iodide (PI)-positive cells [17]. Here, we are further characterizing the progressive increase in osmotic fragility of A549 under serum starvation and presenting additional evidence that the huge ATP level attained by these isolated bursts, which is many folds larger than the “non-lytic” cell secretion, comes from the discharge of whole cellular ATP content.

### 2.1. Serum Deprivation and Osmotic Fragility

While not common, an osmotic imbalance is not rare and occurs in pathophysiological situations such as lung or cerebral edema but also physiologically in the nephron. Hypotonically induced cell swelling is a standard stimulus to study cell volume regulation and a very potent in vitro load to stimulate nucleotide or taurine release and serve as a model to understand the documented release in vivo. Serum starvation is used here as a tool to fragilize cells and simulate pathophysiological conditions akin, for instance, to exposing cells to bacterial LPS. In addition, several transfection protocols or cell cycle synchronization assays use serum-starvation procedures. It is thus important for correct analysis to understand that cells are tremendously fragilized from this serum removal, and this may affect the outcome of the measurements.

**Figure 1:**
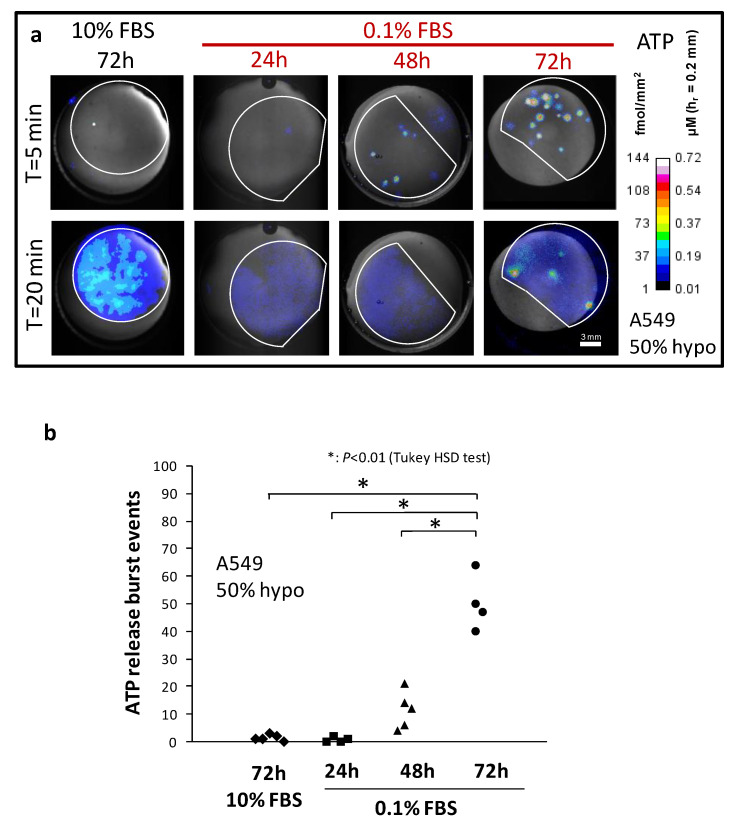
The impact of serum deprivation on osmotic fragility of A549 cells. (**a**) Representative ATP imaging of hypo-stimulated A549 cells grown with 10% FBS (72 h) or 0.1% FBS (24 h, 48 h or 72 h). Cells in 10% FBS 72 h were near confluency (~500 cells/mm^2^) but the cell density at 0.1% FBS 72 h declined ~12-fold. The contour of glass coverslips with cells is outlined in white. Colour-coded images of ATP density, or average [ATP], overlaid on the brightfield-acquired static image (gray) of the hypotonic chamber are shown in response to an acute diminution of extracellular fluid tonicity (50%) at 30 °C. Images at T = 5 and 20 min (medium dilution at T = 0 min) are shown from representative experiments. (**b**) Accumulated lytic events post-50% hypotonic shock (<55 min). Number of A549 cells per coverslip (Mean ± SD): 10% FBS 72 h (128,227 ± 24,235), 0.1% FBS: 24 h (6865 ± 1737), 48 h (8554 ± 2244) and 72 h (10,715 ± 2132).

**Figure 2:**
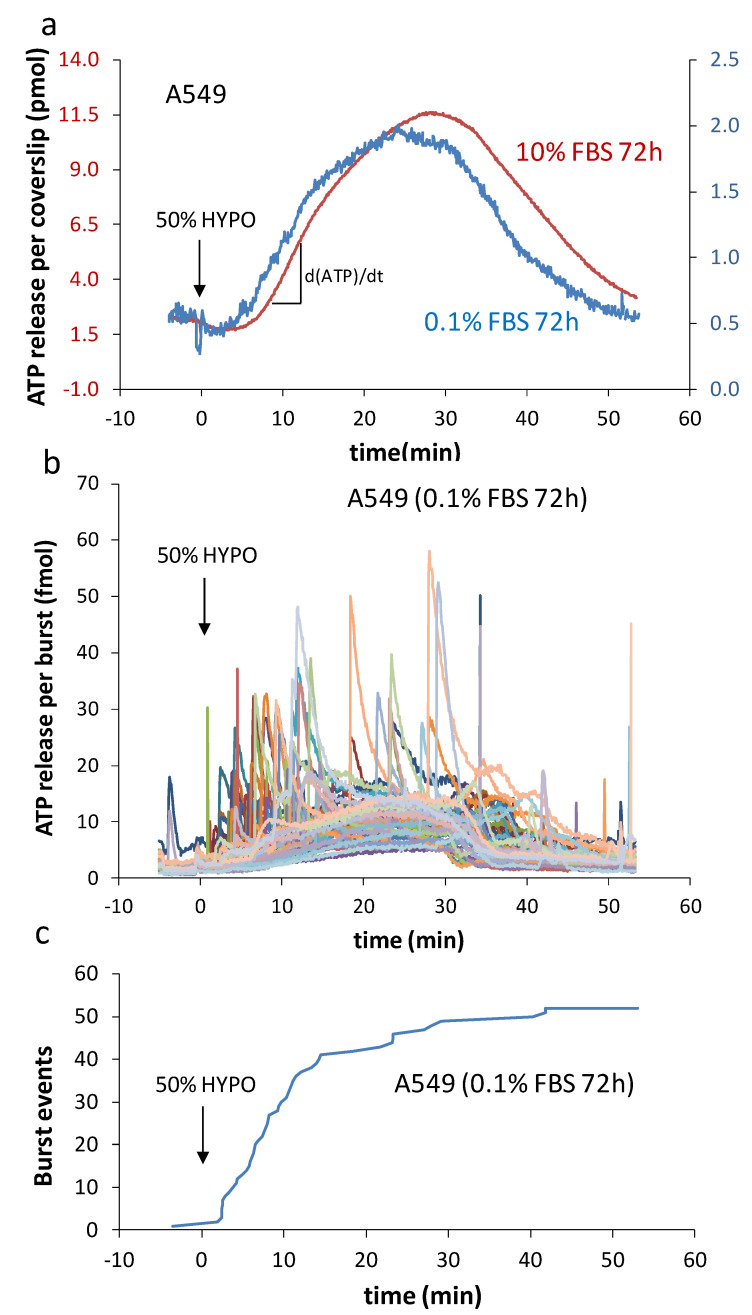
Kinetics of hypo-induced ATP release and burst events. (**a**) Representative accumulated ATP in the chamber over time from coverslip of A549 cells cultured 72 h in 10% FBS (control, red trace, left axis) or 0.1% FBS (blue trace, right axis). The peak efflux rate (around 10 min) for the control trace (72 h, 10% FBS) is d(ATP)/dt = 0.7 pmol/min/coverslip or 7 × 10^−5^ fmol/s/cell and for serum-starved (72 h, 0.1% FBS) is (ATP)/dt = 0.1 pmol/min/coverslip or 2 × 10^−4^ fmol/s/cell. (**b**) ATP level variation over time for individual burst events from a representative 72 h 0.1% FBS test. Note, the slowly rising background comes from ATP diffusing from cells in the vicinity. (**c**) The sum of burst events from the experiment shown in (**b**).

The impact of serum deprivation on A549 osmotic fragility is illustrated in Figure 1a, which shows ATP-dependent luminescence images of hypo-stimulated A549 cells grown with 10% FBS (72 h) or 0.1% FBS (24 h, 48 h or 72 h). There are two types of ATP release responses, highly-localized sudden bursts of ATP secretion (lytic events) and slowly-rising but fainter and widely-spread non-lytic release. It took 48 h of serum starvation for the increase in the number of observed burst events to become noticeable (11 ± 7; 48 h 0.1% FBS) and 72 h to become significantly higher (50 ± 10; 72 h 0.1% FBS) than the control (1.4 ± 1; 72 h 10% FBS), summarized in Figure 1b. This was not a result of a larger cell population per coverslip as low serum stalled cell growth and induced apoptosis resulting in only ~8% of total cell number compared to control after 72 h. Note also that after growing 72 h in low serum, the average proportion of osmolyzed cells in the whole population reached ~0.5% (50 bursts/10,715 cells). The sudden apparition of bright “sparks” of punctual luminescence at high ATP density level within serum-deprived cells population was remarkable (Figure 1a, T = 5 min) compared to the widespread but fainter non-lytic secretion (T = 20 min). Although we observed a lower ATP density level from this non-lytic release originating from serum-deprived cells at T = 20 min (<10 fmol/mm^2^) compared to normal 10% FBS (20–50 fmol/mm^2^, Figure 1a), it was a result of having fewer cells per coverslip due to impaired growth in serum-deprived conditions, not from alteration of ATP secretion intensity (see below for details). Contrary to non-lytic release, which typically started rising after a lag time of ~5 min (see Figure 2a), the bursts occurred almost immediately after imposing hypo-osmotic shock (Figure 1a, T = 5 min). There is uncertainty, however, on the magnitude of the observed lag, as its duration could be smaller in reality. Indeed, intact and progressively swelling cells in these tests likely struggle to accumulate ATP in the extracellular medium above the detection threshold because of the comparatively slower release of ATP through non-lytic pathways against the simultaneous degradation by ecto-ATPase and luciferase, all of these concurring to produce longer lag. Thus, the early occurrence of ATP release bursts associated with cell lysis is most likely an indication that cells experience osmotic swelling early after dilution of the extracellular medium.

### 2.2. Characteristics of Burst Events

Serum deprivation seemingly speeded the hypo-induced accumulation of extracellular ATP over control cells, with a more pronounced difference shown in Figure 2a at 72 h. This faster buildup of ATP in the medium arose from a higher number of cells bursting soon after the medium dilution took place. Notwithstanding this earlier start and the lower quantity of accumulated ATP per coverslip due to reduced cell growth, the response pattern was sensibly similar and showed minimal effect from the low-serum cultivation procedure.

Each individual burst event rapidly reached a peak in <5–10 s (Figure 2b), yielding a very fast ATP secretion rate attaining ~1–5 fmol/s. Assuming the release came from a single cell, which was likely the case, this value was several orders of magnitude faster compared to the average we typically observed from a population of intact cells with dATP/dt = 7 × 10^−5^ fmol/s/cell (Figure 2a). Note that this feeble rate was due in part to the fact that cell volume expansion speed is dependent on diffusion and equilibration of solution tonicity [18] and that the establishment of osmotic gradient was slow in this non-perfused experimental set-up hence a much slower ATP secretion. For comparison, the peak efflux for off-line titrated ATP release with a fast solution replacement perfusion chamber (37 °C) and A549 cells reached ~5 × 10^−3^ fmol/s/cell [19]. While this observed non-lytic efflux is comparatively larger (~70-fold) than the ones reported here, it still remains ~1000-fold lower than the burst event. This astonishing difference in rate (bursts vs non-lytic) further supports the interpretation that these bursts originate from cell lysis. The frequency of these lytic events was higher at the beginning of medium dilution (Figure 2c), with 75% of observed events occurring within the first 15 min, and 90% were observed after 30 min in this representative experiment. Whether serum-deprived or not, the peak of ATP accumulation was typically within 25–30 min, suggesting that lytic events are taking place within the same time frame as non-lytic release and most likely during the swelling phase.

**Figure 3:**
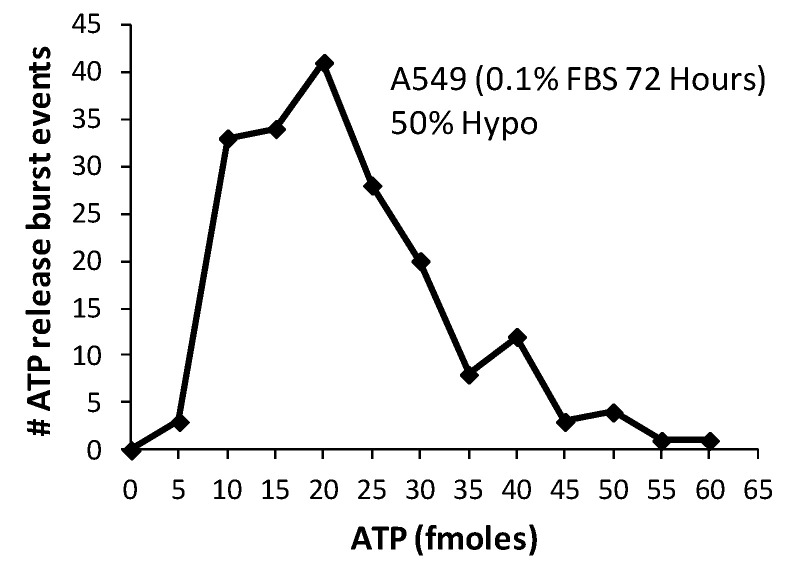
The distribution of burst events. Cumulative 50%-hypo-stimulated ATP release for all burst events in serum-deprived (0.1% FBS, 72 h) A549 cells from 4 independent experiments were ranked. Note that only uniquely quantifiable events were included in the tally (*n* = 188). Bin size: 5 fmol, mean ± sd: 21 ± 13 fmol, mode (peak of distribution): 17 fmol.

An important question to address is whether the individual ATP bursts events we observed were really the product of single-cell lysis. We observed considerable variation in ATP release per burst (see Figure 2b), which seems to contradict the idea that ATP is released by a discrete item, such as a single cell. However, this contradiction is only apparent and rather supports our claim since this kind of data dispersion would actually be expected solely on the basis of the wide distribution of cell volume in a population. We collected the peak values of accumulated ATP (normalized to baseline) from all clearly identified lytic events at 72 h (*n* = 188) and plotted their distribution (Figure 3).

The asymmetry of the burst events distribution with longer right-tail and the mean (21 fmol) being higher than the peak of distribution (mode: 17 fmol) is rightly typical of several reported cell volume population distributions, including NIH-3T3 fibroblasts and vascular smooth muscle cells A10 [20]. An additional variable parameter to account for is the intracellular ATP concentration, typically reported to vary between 1–5 mM. The A549 volume distribution is not known precisely, but we have previously measured volumes ranging between 4–9 pL [21] for a few selected A549 cells (*n* = 6). Omitting the increase in volume from the hypotonic shock and its impact on ATP level, we could estimate the following brackets for cytosolic ATP content: 4 to 45 fmol per cell (4 pL × 1 mM to 9 pL × 5 mM). Our measured ATP burst mode of 17 fmol/cell fall well within these estimated limits and further reinforces our interpretation of the lytic origin of these ATP bursts. In addition, note that the above estimation for average intracellular ATP content is not dissimilar to the limits calculated for the experimentally observed burst events (5 to 60 fmol).

### 2.3. Non-Lytic ATP Release

**Figure 4:**
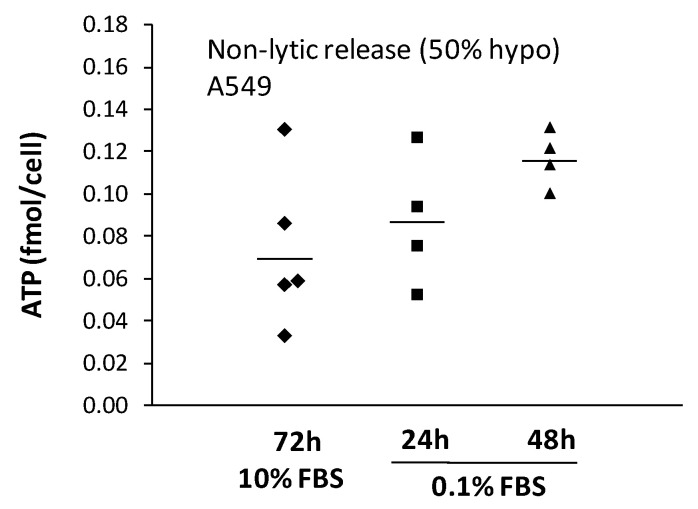
Normalized 50% hypo-stimulated non-lytic ATP release. The sum of ATP secreted by lytic burst events were subtracted from the maximum ATP accumulation per coverslip then normalized with the total cell number. Treatment (mean ± sd in fmol/cell): 10% FBS 72 h (0.07 ± 0.04); 0.1% FBS 24 h (0.09 ± 0.03); 0.1% FBS 48 h (0.12 ± 0.01). ANOVA *p* = 0.14.

We then verified whether serum deprivation affected the level of expelled ATP through the non-lytic pathways. To that end, we subtracted the sum of accumulated ATP for each lytic burst event from the coverslip total ATP accumulation. We had to limit our analysis to 48 h, however, since this approach was unsuited after 72 h of serum deprivation. While lysis concerned only ~0.5% of the cell population during this longer serum deprivation, it provoked too large nucleotide release to precisely distinguish from a non-lytic component. This limited view nonetheless revealed a trend of non-lytic nucleotide release nearly doubling after 2 days of low serum growth (Figure 4). Why we observed a larger nucleotide release in serum-deprived but osmotically resilient cells is unclear but could hint at a more prominent implication of the plasma membrane in that process. Serum starvation is the preferred culture treatment for mRNA-based transfection protocols, but its underlying mechanisms to increase gene expression efficiency are not completely understood. Since fluorescence polarization measurement has revealed that transfection efficiency was boosted 18-fold by a lidocaine-induced membrane fluidity increase [22] and since cellular osmotic fragility also increases with higher membrane fluidity due to lower cholesterol content [23], we could therefore envision that both the increase in lytic events and the increase in the ATP-secretion level are a consequence of the increase in membrane fluidity from the serum starvation. The exact impact of membrane fluidity onto the swelling-induced ATP release pathways, whether lytic or non-lytic, remains unclear and needs further investigation.

In summary, the prolonged period of growth under serum starvation affects only moderately the magnitude of non-lytic release. However, as days of culture progress and as a larger number of A549 cells yield under osmotic stress, the lytic source of ATP release can surpass the one from intact cells. As a result, without proper assessment of lytic release, this could lead to misleading conclusions. Although A549 cells are intrinsically robust against osmotic shock and needed more than 48 h of serum starvation treatment for lysis to increase beyond the naturally occurring level, other cells could be more fragile. For example, ATP imaging of hypotonically challenged mice mammary epithelial cells [24] revealed a series of ATP secretion spikes lasting several minutes after medium dilution similar to the ones we observed in fragile A549 cells. Further, HTC rat hepatoma cells subjected to 30% hypotonic shock presented multiple “point source bursts” when imaged with a real-time chemiluminescent macroscopic imaging configuration [25], highly reminiscent of the lytic events we have reported here. Thus, our short study calls for proper control for the occurrence of cell lysis when exploring osmotically triggered nucleotide release.

## 3. Hemolytic ATP Release in RBCs

The release of ATP by RBCs has been recognized as an important mechanism to increase blood flow in response to the reduced tissue oxygen level. Subsequent activation of vascular endothelial P2Y purinergic receptors stimulates the release of nitric oxide and other mediators of vasodilation, increasing vessel calibre and enhancing blood flow [26,27]. Elevated ATP levels have been found in vivo in venous effluent from exercising forearm muscle [28,29], which were further augmented by exercise performed in hypoxia [26,30]. Both effects, the increase in venous effluent ATP level and vessels dilation in response to low extraluminal O_2_, were only observed when the vessels were perfused with RBCs [26], implicating RBCs in sensing low extraluminal O_2_ and contributing to local blood flow regulation by the release of ATP. Moreover, by modulating the release of ATP, erythrocytes may participate in thermoregulation. There is evidence that the release of ATP from human erythrocytes is sensitive to both an increase and a decrease in temperature and that intravascular infusion of ATP may increase thermal hyperemia in the tissues of the extremities [31]. Thus, regulation of vascular perfusion in response to tissue hypoxia or temperature changes is accomplished, at least in part, through the control of intravascular ATP that is released from RBCs [32,33].

Since mature mammalian RBCs are lacking intracellular organelles, the proposed mechanisms of ATP release included non-exocytotic processes [4], such as conductive release by putative ATP channels CFTR [34], VDAC [35], and Pannexin-1 [36]. However, several earlier studies have found that RBCs hemolysis also contributes to plasma ATP and intravascular hemolysis occurs in vivo under hypoxia and mechanical trauma to RBCs [37,38,39]. Its actual contribution, however, has not been assessed systematically, and several studies attributed the majority of ATP release to cell-regulated processes involving ATP-conducting channels.

### 3.1. Contribution of Hemolysis to ATP Release

**Figure 5:**
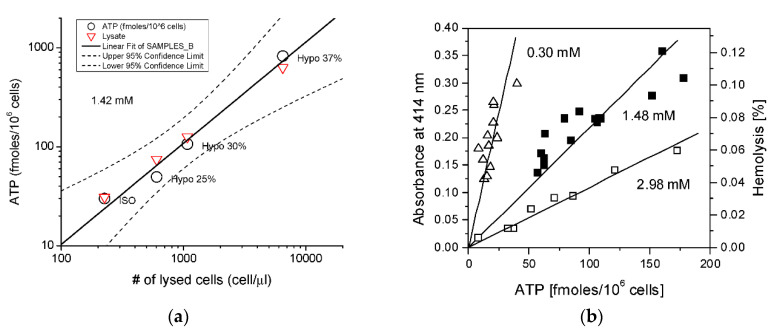
Hypotonic shock-induced ATP release in RBCs. (**a**) Example of paired measurements of hypotonic shock-induced ATP release versus extracellular Hb (expressed as the number of lysed cells/µL) obtained from a single blood donor (O). The solid line is a least square linear fit to ATP release data with 95% confidence bands indicated by dashed lines and correlation coefficient R = 0.988. Red triangles indicate the expected ATP release for a given level of cell lysis and independently determined intracellular ATP concentration of 1.42 mM. (**b**) Relationship between extent of hemolysis (shown as absorbance—left axis, or % hemolysis—right axis) and ATP release induced by hypotonic shock. Paired values of extracellular ATP and free Hb in the supernatant samples were obtained using blood from 3 different donors, indicated by different symbols. In each experiment, RBC suspensions were incubated in either isotonic or hypotonic (20%, 25%, 30%, 35%, or 40%) solution. The slopes of the fitted lines correspond to the intracellular ATP concentration and are indicated on the graph (from [40]).

To determine the contribution of hemolysis to stimulated ATP release from RBCs, we have systematically examined various experimental approaches used by different research groups to measure ATP release and to quantify hemolysis of RBCs [40]. Paired measurements of ATP and free hemoglobin (Hb) in each sample of RBC supernatants were chosen as the most reliable and reproducible approach. With this method, we have shown that basal and hypotonic shock-stimulated ATP release correlated tightly with extracellular Hb from lysed cells, as shown in Figure 5a (black circles). To verify if the observed ATP release matched the levels expected exclusively from cell lysis, the intracellular ATP content was measured independently in cell lysates prepared by Triton-X treatment of the same batch of blood. The expected ATP release was then calculated from corresponding numbers of lysed cells under isotonic (Iso) and hypotonic conditions (red triangles). They matched perfectly with the observed release, demonstrating that hypo-induced ATP release in RBCs is entirely attributable to cell lysis, as shown in Figure 5a. The same tight relationship was observed for blood batches obtained from different donors, as shown in Figure 5b. The figure also shows that cell batches of higher osmotic fragility (higher % of hemolysis vs released ATP) are the ones that have lower intracellular ATP content, consistent with RBCs fragility dependence on their metabolic status [41].

### 3.2. Luminescence Imaging Demonstrates ATP Release Exclusively from Lysing Cells

**Figure 6:**
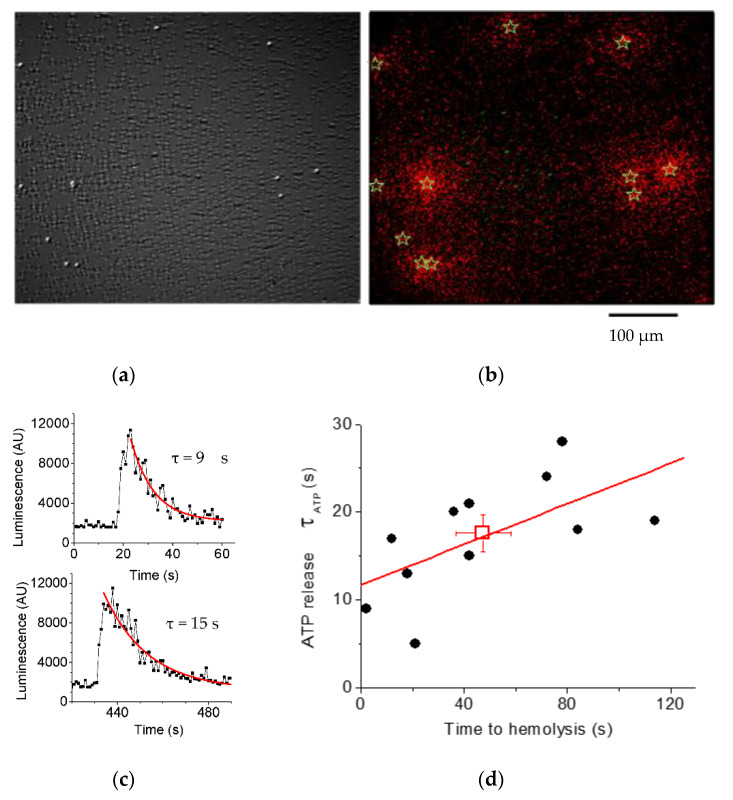
Luminescence imaging of ATP release from RBCs. (**a**) IR-DIC image of RBCs attached to the glass substrate with lysed cells visible as white spots acquired 25 min after 20% hypotonic shock. (**b**) Overlay of image (**a**) and cumulative ATP-dependent luminescence (in red) observed during the entire experiment. Note that regions of ATP release coincide with lysed cells (marked by stars). (**c**) Two examples of luminescence time-course responses due to lysis of single RBCs in the same experiment. The characteristic time of ATP release (τ) was determined by exponential fitting of the decaying phase of the luminescence signal (red line). (**d**) A plot of the single-cell ATP release time τ and the corresponding duration of Hb release (time delay to hemolysis) for the same experiment. Red line—linear fit to the data, and the red square represents the Average ± SD of the release time for ATP (17.6 ±2.1 s) and Hb (47.6 ±10.6 s). (Adapted from [40]).

The primary role of hemolysis in hypotonic shock-induced ATP release was confirmed more directly by real-time simultaneous ATP-imaging and infrared (IR) differential interference contrast (DIC) imaging of substrate-attached RBCs. With luciferin-luciferase (LL) present in the extracellular solution, these experiments identified single ATP-releasing cells and revealed that only lysing cells contributed to the release. This is illustrated in Figure 6a,b, which shows that following osmotic stimulation, the ATP release was seen as a flash of ATP-dependent LL luminescence around the cells that subsequently disappeared from the image due to leakage of their Hb content (cell ghosting). Ghosting occurred within up to 120-s after the peak of the ATP release, indicating that Hb release proceeds significantly slower compared to ATP release. This is demonstrated in Figure 6c, which shows examples of single-cell ATP release spikes and their decay time (τ), while Figure 6d shows a plot of τ vs. duration of Hb release (time to ghosting). Cell ghosting due to Hb leakage takes, on average, ~3-times longer than ATP release, which is similar to the ratio of diffusion coefficients for ATP compared to that of Hb in water (D_ATP_/D_Hb_ = 3.8), supporting the view that release of Hb and ATP proceeds through the common pathway.

Thus, the study demonstrated that hemolysis is likely the only mechanism of ATP release induced by hypotonic shock in RBCs. Unexpectedly, the same tight relationship between ATP release and % hemolysis was seen with other stimuli tested (shear stress, hypoxia), with no evidence of regulated release from intact cells [40]. This finding has important consequences regarding the physiological roles of intravascular hemolytic ATP release. RBCs form a non-homogenous population, which contains cells at different stages of their life cycle, including senescent cells that are in a prolytic state before removal from circulation [42]. Ageing RBCs show the accumulation of membrane microdefects, the reduced level of the cell membrane stomatin and sialoglycoproteins, the translocation of phosphatidylserine to the outer leaflet of the cell membrane, which all contribute to altered membrane mechanical and electrical properties and increased cell fragility. Therefore, senescent RBCs constitute an easily accessible pool of ATP for local purinergic signalling. Importantly, besides natural ageing, other processes that control RBC susceptibility to lysis under stress and hypoxia will modulate ATP release, discussed in [33,42].

## 4. Ultrasound and Microbubble-Induced ATP Release for Enhancing Cancer Immunotherapy

Immunotherapy is now successfully complementing radiotherapy, surgery and chemotherapy in our arsenal against cancer [43]. Immune checkpoint blockade therapy with antibodies targeting CTLA-4 and PD-1 have provided durable clinical benefits, including complete tumour rejection [44]. However, these treatments can be associated with severe toxicities and, unfortunately, are curative only in subsets of patients [45]. It is becoming apparent that the presence of non-redundant immunosuppressive pathways, as well as physico-chemical characteristics of the tumour microenvironment (TME), limit the activity of cancer immunotherapies. Novel strategies and approaches that address these barriers could help increase the efficacy of cancer immunotherapy. Understanding the role and targeting the therapeutic potential of purinergic signalling for cancer immunotherapy is an active and promising area of research. ATP and its downstream metabolite Ado are important immunomodulators in the TME, and their effects on antitumor immune responses have been recently and extensively reviewed [46,47,48,49]. For instance, targeting CD39 and CD73, two key rate-limiting enzymes that, respectively, degrade ATP into ADP and AMP and AMP into Ado with antibodies or pharmacological inhibitors, have been shown to cause robust anticancer immune responses in rodents and are currently undergoing clinical trials [50,51]. Small molecule drug candidates inhibiting P1 and P2 purinergic receptors are also showing therapeutic promise [47]. However, the systemic administration of these powerful inhibitors can lead to harsh side effects [51], which could be alleviated by a local potentiation of these active compounds. Interestingly, ultrasound (US)-targeted microbubble (MB) cavitation (UTMC) offers an externally triggered, spatially controlled and image-guided approach that is promising for local drug release [52,53] and for improving antibody extravasation and efficacy [54,55,56,57]. However, these exciting results overshadow the fact that the biological effects and signalling pathways stimulated by MB–cell interactions are still poorly understood. In particular, the prominent role of purinergic signalling following UTMC was only recently discovered [58] and has been studied by a single team of investigators. In this section, we will briefly review the current knowledge on MB–cell interactions and more specifically describe in vitro and in vivo ATP release kinetics following UTMC therapy. Our recent data support the therapeutic potential of UTMC for enhancing cancer immunotherapy in solid tumours.

### 4.1. Microbubbles for Imaging and Therapy

MBs are micron-sized gas-filled spheres stabilized by a shell (lipid, polymer or proteins) that can be injected intravenously as blood tracers and act as a contrast agent for clinical US imaging. As they travel in the bloodstream, MBs are imaged using dedicated contrast-mode pulsing sequences that cancel out tissue background, thus allowing quantification of tissue perfusion. MB cavitation is generally harmless when excited with low energy US pulses. Indeed, MBs have been used clinically for decades in diagnostic applications, and their safety has been clearly established in this context [59]. When stimulated by higher amplitudes and/or longer US pressure wave durations, MBs undergo stronger oscillations that locally amplify the thermal and mechanical effects of the US. In this scenario, MBs become theranostic agents that act simultaneously as imaging and therapeutic agents, in which US imaging is used to guide the positioning of the therapeutic US beam and to track MB replenishment between therapeutic pulses. For very high levels of energy, which can be obtained with high power dedicated equipment (High Intensity Focused Ultrasound), thermal effects can be achieved and even lead to the generation of light [60]. On the other hand, mechanical effects can be generated with lower energy requirements (shorter pulse lengths < hundreds of μs; low duty cycle ~0.1%) and intermediate pressure ranges (mechanical index < 1.9). For instance, vasodilation can be stimulated by clinical scanners with repeated stimulations at diagnostic pressures [58,61]. Several drugs, plasmid DNA and miRNA delivery have been successfully implemented using clinical scanners [62,63,64,65]. However, many therapeutic applications of UTMC seem to benefit from longer pulse durations, which sustain the cavitation activity [66,67,68,69]. Exhaustive reviews of therapeutic applications being investigated for MB cavitation can be found in [70,71].

### 4.2. Sonoporation

One interesting type of MB–cell interaction is the mechanical rupture of membrane integrity by an oscillating MB, causing the formation of membrane pores, a process termed “sonoporation”. Both stable and inertial cavitation can cause the formation of current-conducting pores detectable by the patch-clamp technique [72]. During membrane poration, membrane-impermeable molecules (such as propidium iodide, often used as a model drug) have been observed to gain access to the cell cytoplasm [73,74]. In vitro, pores may or may not reseal, the latter leading to cell death [75]. Pore resealing has been observed to occur within 1 min, depending on the pore size and is calcium-dependent [76]. If calcium ions are present in the extracellular medium, sonoporation can lead to a calcium influx and generate a calcium wave in adjacent cells [77]. Sonoporation has been shown to be related to the mechanical shear stress generated by the MB oscillations. For an MB positioned next to the membrane, the MB excursion radius provides a threshold quantity beyond which pore formation will occur (~0.7 μm, but frequency-dependent) [73,74]. Porating cell membranes with UTMC in vitro is relatively straightforward, as the US can be pulsed repeatedly, and new MBs can be introduced as cavitation nuclei. However, finding the balance to generate pores that reseal and maintain high cell viability remains challenging. For cultured cells, a recent study reported that most sonoporated cells did not survive sonoporation [78]. On the other hand, there are reports of a combination of US parameters that allow reaching a high sonoporation rate while maintaining cell viability for plated [79] and suspended [75] cells. Dosing of MB activity will be an important factor to consider in the clinical translation of these techniques, especially in sensitive organs such as the brain. Indeed, petechiae [80] and hemolysis [81] can be cited as examples of overt membrane rupture and can occur with harsh and sustained UTMC therapy. These effects need to be weighted in the risk-benefit analysis for each therapeutic application. Cavitation detectors have been developed to mitigate and control MB activity [82]. The reader can find additional information on sonoporation here [71].

### 4.3. Beyond Sonoporation

MB–cell interactions and cell signalling beyond the biophysical creation of a pore are not well understood to date, and further studies will be needed to unravel the complexities of cell signalling and cell interactions following UTMC. Within the sonoporated cell, the downstream effects of sonoporation are being unravelled: calcium influx [83], membrane depolarization [84], F-actin remodelling [85], cell blebbing [86], and the activation of heat shock protein 70 [87], leading to late apoptosis even in cell initially surviving sonoporation. MB oscillations have been shown to enhance endocytosis and pinocytosis in vitro [88] and in vivo [89]. The long-term fate of sonoporated cells has been evaluated and is suggesting that sonoporation is very stressful for cells in vitro and may jeopardize their long-term survival [87]. However, successful cellular transfections have been described in vitro and in vivo [63,65,90], suggesting that cells can survive a sonoporation event. It seems logical to postulate that the understanding of intercellular signalling following sonoporation will be a key in future developments in this field. Our understanding of intercellular signalling has evolved from that related mostly to vascular biology to other pathways, including inflammatory signalling. We now have evidence that UTMC: (1) can cause eNOS phosphorylation and nitric oxide generation in endothelial cells, leading to smooth muscle cell relaxation causing vasodilation [58,68]; (2) can affect endothelial cell–cell contact through yet unidentified signalling mechanisms [74]; (3) can enhance the expression of vascular inflammation markers (ICAM) in the brain within 1 h post sonication and elevations in heat shock protein 70, interleukin-1, interleukin-18, and tumour necrosis factor-α indicative of a sterile inflammatory response in the brain [91].

### 4.4. Microbubble-Driven ATP Release

The ability of MB oscillation to release ATP is a relatively recent observation [58]. It was shown that UTMC with a clinical scanner (1.3 MHz, 1.3 MI, SONOS) for 10 min caused a 10-fold increase in muscle blood flow in a mouse model and the local release of nitric oxide [68,92]. This provascular response was found to be mediated by a local release of ATP, which was still detectable by bioluminescence imaging 24 h post-treatment [58]. In separate in vitro experiments, ATP was found to originate from endothelial cells and red blood cells, which are known ATP reservoirs in the vasculature. Fluorescent dyes added during or after UTMC demonstrated that sonoporation had occurred. Most interestingly, endothelial cells continued to release ATP 20 min after sonoporation, indicative of delayed cell death/apoptosis contributing to the sustained release observed in vivo. Whether Pannexin-1 is also involved, as suggested in [93], requires further study. Thus, UTMC-mediated ATP release could involve multiple mechanisms and reservoirs, including acute and delayed release resulting from sonoporation with distinct released amounts and kinetics.

### 4.5. Kinetics of ATP Release In Vitro

**Figure 7:**
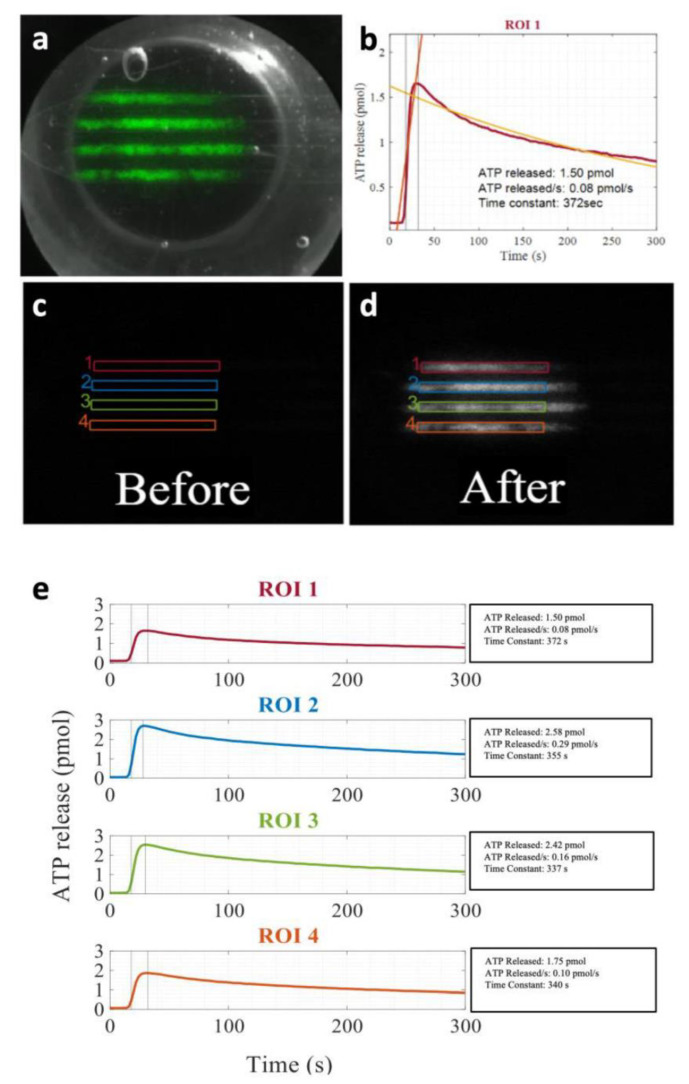
UTMC-induced ATP release in mammary carcinoma 4T1 cells. (**a**) An overlay of the peak ATP signal (green) on top of a brightfield image showing the US probe in the background. The image shows four microfluidic flow channels on the PDMS chip with plated cells. (**b**) Typical ATP release kinetics in a region of interest (ROI) and signal processing with the signal coloured in red, the linear fit for the ATP release in orange, and the exponential fit in yellow; ATP image (**c**) before and (**d**) after the US pulse in the 4 ROIs analyzed; (**e**) ATP release kinetics with analyzed values for the ATP released, ATP release peak rate, and release time constant.

We investigated the effect of the number (#) of cycles and US pressure on ATP release kinetics in vitro to better understand the ATP release mechanisms. Mammary carcinoma 4T1 cells were cultured in custom PDMS chips (see Methods) and incubated with MB by inverting the chip upside down to allow MB to float into contact with the cells. Different US pressures and #cycles were delivered using a single element US probe at 1 MHz (Figure 7a).

**Figure 8:**
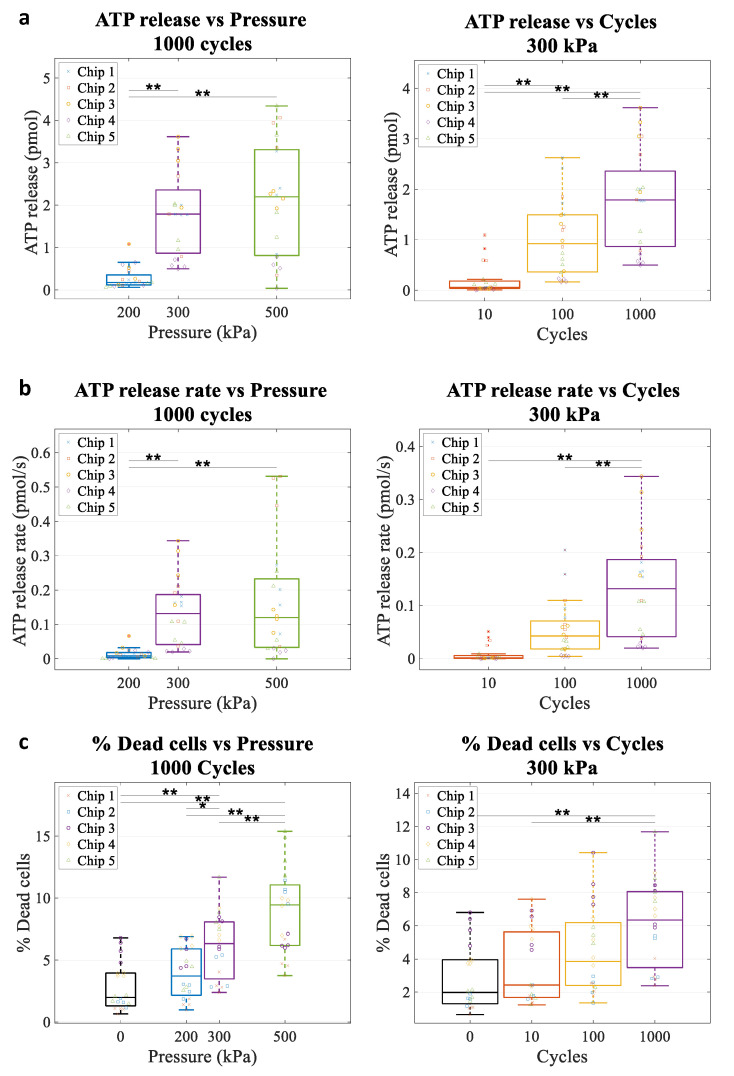
The effect of US pressure and # cycles on the ATP release and % dead cells in 4T1 cells. (**a**) ATP release amount, (**b**) ATP release rate (peak)**,** and (**c**) % dead cells following UTMC therapy. Boxplots are represented as median, 25th–75th percentiles, and range (excluding outliers > 1.5 × interquartile range); *n* = 20 (5 repeats, 4 channels per chip). * *p* < 0.05; ** *p* < 0.01 (Tukey HSD post hoc test).

ATP release kinetics were recorded using a bioluminescence imaging system [16] (Figure 7a–d). We found that ATP release was triggered by US delivery in an area delimited by the probe beam (Figure 7a). Across all pulses, ATP release varied between 0.03 pmol and 2.7 pmol. This is similar to ATP released by a strain of 5% to 9% [16]. ATP release rate varied between undetectable levels and up to 0.24 pmol/s. Both pressure and #cycles had an effect on ATP release amount (Figure 8a) and ATP release rate (Figure 8b) (all *p* < 0.001, one-way ANOVA). Increasing pressure and # cycles caused significant increases in ATP release amount and rate that were significant based on multiple comparisons between groups. These observations are consistent with an increase in cavitation efficiency/activity increasing pulse length and pressure. In separate experiments, propidium iodide and calcein-AM were added 5 min after US exposure to assess cell viability. Percent of dead cells also increased with pressure and #cycles (Figure 8c).

**Figure 9:**
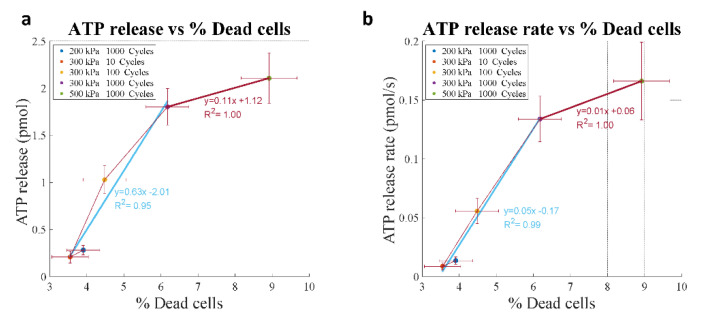
The relationship between % dead cells and ATP release in 4T1 cells. Graphs show ATP release amount (**a**) and rate (**b**), data are the Average ± SE. Both parameters increased with % dead cells, indicating that permanent sonoporation causing cell death was a major ATP release mechanism following UTMC.

When plotting ATP release against % dead cells, we could identify two regions (Figure 9): (1) a region with lower ATP release (~1 pmol) and a low percentage of dead cells (2% increase compared to control), corresponding to lower energy pulses (blue line); (2) a region with a marked increase in ATP release (up to 2.5 pmol) but with a higher percentage of dead cells (6% increase compared to control), corresponding to higher energy pulses (red line). Interestingly, these two regions had different slopes of ATP release as a function of % dead cells, which differed by a factor of 6, suggesting different release mechanisms, respectively, non-lethal (pores that reseal, in blue) and lethal (permanent poration, in red). Similar observations were found for ATP release rate against the percentage of dead cells in which two regions also appeared: (1) a region with slower release speed (<0.14 pmol/s), associated with a lower percentage of dead cells and corresponding to lower energy pulses (blue line); (2) a region with higher release speeds (>0.14 pmol/s), associated with a higher percentage of dead cells and corresponding to high energy pulses. These observations seem consistent with an increase in the pore size and number with increasing US cavitation.

### 4.6. ATP Release In Vivo in Muscle and in Tumors

**Figure 10:**
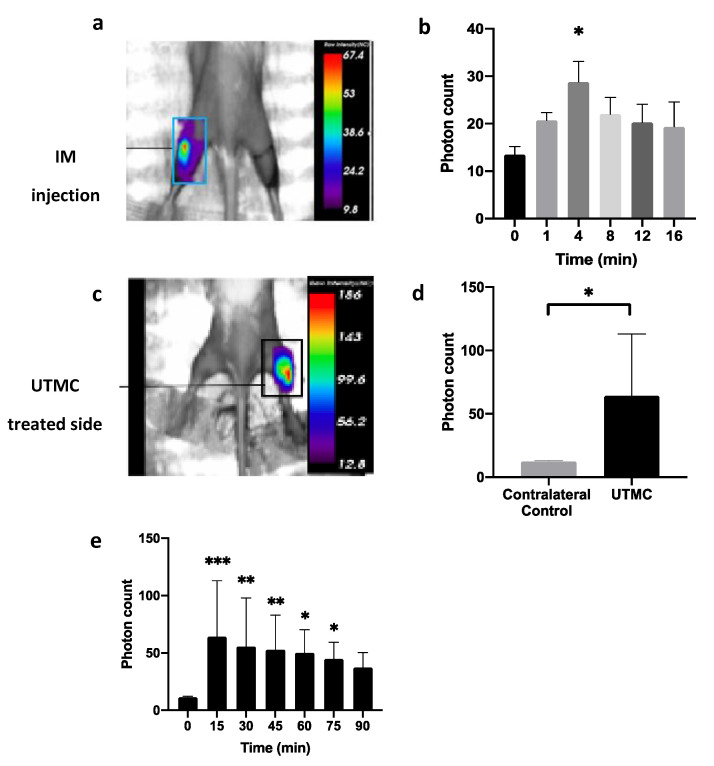
Extracellular ATP signal quantification in mice muscle tissue using in vivo bioluminescence imaging. (**a**) ATP-dependent bioluminescence signal (T = 4 min) following intramuscular injection of bolus ATP (20 µL bolus, 250 μM, at T = 0 min); (**b**) eATP kinetics following injection shown in (a) (*n* = 3, * *p* < 0.05 vs T = 0 min, Dunn’s test); (**c**) eATP signal measured by optical imaging 15 min post-UTMC. (**d**) eATP signal in treated (UTMC) and contralateral side at T = 15 min (*n* = 6, * *p* < 0.05, Wilcoxon test); (**e**) eATP kinetics after UTMC treatment (*n* = 6, * *p* < 0.05, ** *p* < 0.01, *** *p* < 0.001 vs T = 0 min, Dunnett *post hoc* test). Data represented as (Average ± SD).

We then tested if ATP could be released in muscle using UTMC using whole-body luminescence imaging. We first performed a positive control experiment in which a 20 μL bolus of ATP (250 μM) was directly injected intramuscularly (Figure 10a). The ATP-dependent bioluminescence signal peaked at 4 min (28.8 ± 4.3 photons) but was not different from the baseline value at 8 min post-injection (~20 photons). We then exposed the right hindlimb of shaved mice to UTMC (1 MHz, 5000 cycles, 800 kPa), repeated every 5 s for 10 min. The therapeutic transducer was positioned perpendicularly to an imaging clinical scanner (15L8 probe, Acuson Sequoia, Siemens) used in CPS mode (7 MHz, MI = 0.2) to guide therapy. MBs (Definity) were infused at a constant rate of 4 µL/mL using a syringe pump. The contralateral side was used as a control (Figure 10b). After UTMC, a high level of extracellular ATP (eATP) signal was detected in the muscle. The signal was 5.5 folds greater in the treated side (64.2 ± 48.6 photons) compared to the non-treated side (11.6 ± 1.2 photons) at 15 min, and it persisted up to 75 min post-treatment. This data supported that UTMC can release high amounts of ATP in vivo at concentrations higher than 250 μM, as supported by the amplitude and duration of the bioluminescence signal compared to direct intramuscular injection. This ATP release was triggered and restricted by the US beam since there was no signal in the contralateral hindlimb.

**Figure 11:**
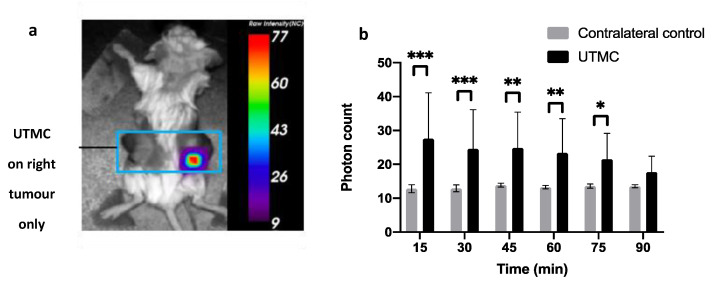
The eATP signal after UTMC in 4T1 xenografted tumors in mice. (**a**) eATP signal measured by optical imaging 15 min post-treatment; (**b**) time course of eATP signal (photon count) after UTMC therapy in treated side versus non-treated side (*n* = 7, * *p* < 0.05, ** *p* < 0.01, *** *p* < 0.001, Sidak test). Data represented as the Average ± SD.

Finally, in 4T1 xenografted bilateral tumours in mice, bioluminescence imaging revealed an eATP signal in the tumour after UTMC treatment (Figure 11a). The mean photon count was 2.2 times greater in the treated side (27.5 ± 13.5 photons) compared to the non-treated side (12.8 ± 1.2 photons) at 15 min, and the signal persisted up to 75 min post-US + MB (21.5 ± 7.6 photons) (Figure 11b). Based on our data in muscle, the ATP release was also higher than 250 μM in the tumours. To the authors’ knowledge, this is the first report of in-vivo ATP release kinetics following UTMC therapy in tumours.

In summary, in this section, we presented a general overview of the ability of MB cavitation to cause ATP release in the form of an image-guided, spatially targeted theranostic approach in cell culture, in muscle and in xenografted tumours. The mechanisms involved and therapeutic applications (vasodilation, damage-associated molecular pattern (DAMP) signalling) are only starting to be understood. It is likely that ATP is released from cells near the cavitating MB (likely RBC and endothelial cells) and that a mechanism allows sustaining this release in time. We are currently testing the potential of this approach for immune stimulation, which will possibly require strategies to capitalize on the local ATP release and prevent its conversion into immunosuppressive adenosine.

## 5. Concluding Remarks

Investigating ATP release due to cell lysis might be warranted. As quantified in Section 2, the level of lytic-induced ATP release, while resulting in the death of one cell, totalizes the nucleotide release of several hundreds of intact cells and, as such, possesses a potent purinergic signalling capability to target a wide range of cells. This is well illustrated by intravascular hemolytic ATP release from RBCs, where lysis of a single erythrocyte will result in ATP concentration of ~1 μM sufficient for triggering purinergic control of blood flow within 2–10 mm long segment of a microcapillary with a diameter comparable to RBC size (7 μm) [33]. In certain pathophysiological conditions associated with prominent cells fragilization, the lytic sources of ATP and their downstream signalling molecules (adenosine) could be the most abundant and principal effectors. Potent purinergic effects due to lytic nucleotide release could be utilized therapeutically, as illustrated in Section 4, where we quantified ATP release kinetics in vitro and in vivo following ultrasound-mediated microbubble cavitation. Understanding the mechanisms and reservoirs of ATP release using an image-guided approach could lead to novel therapeutic opportunities such as immunomodulation in the tumoral microenvironment.

## 6. Methods

### 6.1. Methods for Section 2

Human lung carcinoma A549 were seeded with High Glucose DMEM supplemented with 10% FBS or 0.1% FBS, 2 mM L-glutamine, 50 U/mL penicillin-G and 50 μg/mL streptomycin sulphate at 37 °C and 5% CO_2_ on 15-mm diameter glass coverslips for 24 to 72 h. On the day of the experiment, cells on the coverslip were placed in a glass-bottomed chamber and bathed at 30 °C in 500 µL of LL-DMEM (Luciferin-Luciferase ATP detection reagents in phenol red-free DMEM). Before the experiment, the chamber with cells seeded on a coverslip was left in an incubator at 37 °C and 5% CO_2_ for 15–20 min. This allowed for a reduction of the ATP level induced by mechanical stimulation during coverslip transfer. During the experiment, the ATP release was stimulated by adding 500 µL of LL-H_2_O (including 1 mM of MgCl_2_ and CaCl_2_), yielding ~50% hypo final. ATP-derived luminescence was collected with a custom wide FOV imaging system using an EMCCD camera and a 3:1 reducing optical system. The ATP calibration technique and more experimental details can be found in [16]. To minimize fluid perturbation, LL-H_2_O solution was slowly added in the chamber over an area free of cells. Series of 5-s duration images were recorded during 60 min.

### 6.2. Methods for Section 4

*PDMS cell culture chips*. Microfluidic chips were manufactured using Polydimethylsiloxane (PDMS) (Sylgard 184, Dow). PDMS was mixed at a 10:1 ratio of PMDS to curing agent, poured onto the moulds and degassed in a vacuum. The degassed PMDS in the moulds was then cured at 70 °C for 90 min. The two halves of the chips were bonded together by exposing the faces to atmospheric plasma for 30 s.

In vitro *experiments*. Mammary carcinoma 4T1 cells were seeded in custom PDMS chips (10^5^ cells/chip, each flow channel 700 μm × 700 μm × 17 mm) to reach 90% confluency overnight and incubated upside down with microbubbles (Definity) to allow MB floating up to the cells (5 min) at a concentration of 6 MB/cell. Three different US pressures (200, 300, and 500 kPa) and three number of cycles (10, 100, and 1000 cycles) were tested using single US pulses, delivered using a single element US probe (1 MHz, A303S, 0.5 Inch, Olympus). These experiments were conducted under static conditions (no flow). A bioluminescent imaging system (Evolve 512, Photometrics) and a LL assay (FLAAM Sigma-Aldrich, see methods in [16]) were used to quantify ATP release kinetics after US treatment. ATP image sequences were analyzed using a custom MATLAB script. Four rectangular ROIs of equal area were drawn in flow channels subjected to an US. To obtain the amount of ATP in each frame, the noise was first subtracted from the image. Then, the signal was integrated over the ROI and multiplied by a calibration factor determined in [16]. The total ATP release was calculated as the peak ATP signal minus the average pre-US value. The ATP release increased with pressure and # cycles, which is consistent with an increase in cavitation activity. In separate experiments, Calcein-AM (4 µg/mL Thermo Fisher) and propidium iodide (PI, 25 µg/mL Thermo Fisher) were, respectively, added 5 and 30 min after UTMC to assess cell viability after US exposure.

In vivo *protocols.* Wild-type BALB/c mice (6 weeks age, Charles Rivers Laboratories, Senneville, Canada) were anesthetized using 2% isoflurane and depilated. Luciferase and microbubbles were injected using a polyethylene catheter inserted in the jugular vein. 4T1 cells were used as a tumour model for both in vitro and in vivo studies. The treatment was performed when tumours reached a size between 300–400 mm^3^. Whole-body bioluminescence assays were performed using a small animal imager (OptixMX2, General Electric) at a resolution of 2 mm with an integration time of 5 s. Luciferin (3 mg) was administered intraperitoneally (IP) and luciferase (270 µg) intravenously (jugular vein) before UTMC treatment.

## Data Availability

Data are available from the corresponding author upon specific request.

## References

[1] Zimmermann H. (2015). Extracellular ATP and other nucleotides—Ubiquitous triggers of intercellular messenger release. Purinergic Signal.

[2] Mikolajewicz N., Mohammed A., Morris M., Komarova S.V. (2018). Mechanically-stimulated ATP release from mammalian cells: Systematic review and meta-analysis. J. Cell Sci..

[3] Lazarowski E.R. (2012). Vesicular and conductive mechanisms of nucleotide release. Purinergic Signal.

[4] Praetorius H.A., Leipziger J. (2009). ATP release from non-excitable cells. Purinergic Signal.

[5] Locovei S., Bao L., Dahl G. (2006). Pannexin 1 in erythrocytes: Function without a gap. Proc. Natl. Acad. Sci. USA.

[6] Chiu Y.-H., Ravichandran K.S., Bayliss D.A. (2014). Intrinsic properties and regulation of Pannexin 1 channel. Channels.

[7] Taruno A. (2018). ATP Release Channels. Int. J. Mol. Sci..

[8] Taruno A., Vingtdeux V., Ohmoto M., Ma Z., Dvoryanchikov G., Li A., Adrien L., Zhao H., Leung S., Abernethy M. (2013). CALHM1 ion channel mediates purinergic neurotransmission of sweet, bitter and umami tastes. Nature.

[9] Cotrina M.L., Lin J.H.-C., Alves-Rodrigues A., Liu S., Li J., Azmi-Ghadimi H., Kang J., Naus C.C.G., Nedergaard M. (1998). Connexins regulate calcium signaling by controlling ATP release. Proc. Natl. Acad. Sci. USA.

[10] Reddy M.M., Quinton P.M., Haws C., Wine J.J., Grygorczyk R., Tabcharani J.A., Hanrahan J.W., Gunderson K.L., Kopito R.R. (1996). Failure of the Cystic Fibrosis Transmembrane Conductance Regulator to Conduct ATP. Science.

[11] Boudreault F., Grygorczyk R. (2002). Cell swelling-induced ATP release and gadolinium-sensitive channels. Am. J. Physiol. Cell Physiol..

[12] Colombini M. (2012). VDAC structure, selectivity, and dynamics. Biochim. Biophys. Acta (BBA) Biomembr..

[13] Okada Y., Okada T., Islam R., Sabirov R. (2018). Molecular Identities and ATP Release Activities of Two Types of Volume-Regulatory Anion Channels, VSOR and Maxi-Cl. Curr. Top. Membr..

[14] Matsuura H., Kojima A., Fukushima Y., Xie Y., Mi X., Sabirov R.Z., Okada Y. (2021). Positive Inotropic Effects of ATP Released via the Maxi-Anion Channel in Langendorff-Perfused Mouse Hearts Subjected to Ischemia-Reperfusion. Front. Cell Dev. Biol..

[15] Mim C., Perkins G., Dahl G. (2021). Structure versus function: Are new conformations of pannexin 1 yet to be resolved?. J. Gen. Physiol..

[16] Tan J.J., Ponomarchuk O., Grygorczyk R., Boudreault F. (2019). Wide field of view quantitative imaging of cellular ATP release. Am. J. Physiol. Cell Physiol..

[17] Grygorczyk R., Boudreault F., Tan J.J., Ponomarchuk O., Sokabe M., Furuya K. (2019). Mechanosensitive ATP release in the lungs: New insights from real-time luminescence imaging studies. Curr. Top. Membr..

[18] Ponomarchuk O., Boudreault F., Orlov S.N., Grygorczyk R. (2016). Calcium is not required for triggering volume restoration in hypotonically challenged A549 epithelial cells. Pflügers Archiv..

[19] Boudreault F., Grygorczyk R. (2004). Cell swelling-induced ATP release is tightly dependent on intracellular calcium elevations. J. Physiol..

[20] Halter M., Elliott J.T., Hubbard J.B., Tona A., Plant A.L. (2009). Cell volume distributions reveal cell growth rates and division times. J. Theor. Biol..

[21] Boudreault F., Grygorczyk R. (2004). Evaluation of rapid volume changes of substrate-adherent cells by conventional microscopy 3D imaging. J. Microsc..

[22] Nozaki T., Ogawa R., Feril L.B., Kagiya G., Fuse H., Kondo T. (2003). Enhancement of ultrasound-mediated gene transfection by membrane modification. J. Gene Med..

[23] Bernecker C., Köfeler H., Pabst G., Trötzmüller M., Kolb D., Strohmayer K., Trajanoski S., Holzapfel G.A., Schlenke P., Dorn I. (2019). Cholesterol Deficiency Causes Impaired Osmotic Stability of Cultured Red Blood Cells. Front. Physiol..

[24] Furuya K., Sokabe M., Grygorczyk R. (2014). Real-time luminescence imaging of cellular ATP release. Methods.

[25] Feranchak A.P., Lewis M.A., Kresge C., Sathe M., Bugde A., Luby-Phelps K., Antich P.P., Fitz J.G. (2010). Initiation of Purinergic Signaling by Exocytosis of ATP-containing Vesicles in Liver Epithelium. J. Biol. Chem..

[26] Dietrich H.H., Ellsworth M.L., Sprague R.S., Dacey R.G. (2000). Red blood cell regulation of microvascular tone through adenosine triphosphate. Am. J. Physiol. Heart Circ. Physiol..

[27] Ellsworth M.L., Sprague R.S. (2012). Regulation of blood flow distribution in skeletal muscle: Role of erythrocyte-released ATP. J. Physiol..

[28] Ellsworth M.L., Forrester T., Ellis C., Dietrich H.H. (1995). The erythrocyte as a regulator of vascular tone. Am. J. Physiol..

[29] Forrester T. (1972). An estimate of adenosine triphosphate release into the venous effluent from exercising human forearm muscle. J. Physiol..

[30] González-Alonso J., Olsen D.B., Saltin B. (2002). Erythrocyte and the Regulation of Human Skeletal Muscle Blood Flow and Oxygen Delivery: Role of circulating ATP. Circ. Res..

[31] Kalsi K.K., Chiesa S.T., Trangmar S.J., Ali L., Lotlikar M.D., González-Alonso J. (2017). Mechanisms for the control of local tissue blood flow during thermal interventions: Influence of temperature-dependent ATP release from human blood and endothelial cells. Exp. Physiol..

[32] González-Alonso J., Kalsi K.K. (2015). The ubiquitous ATP molecule: Could it be the elusive thermal mediator igniting skin perfusion and sweating in the heat-stressed human?. J. Physiol..

[33] Grygorczyk R., Orlov S.N. (2017). Effects of Hypoxia on Erythrocyte Membrane Properties—Implications for Intravascular Hemolysis and Purinergic Control of Blood Flow. Front. Physiol..

[34] Sprague R.S., Ellsworth M.L., Stephenson A.H., Kleinhenz M.E., Lonigro A.J. (1998). Deformation-induced ATP release from red blood cells requires CFTR activity. Am. J. Physiol..

[35] Sridharan M., Bowles E.A., Richards J.P., Krantic M., Davis K.L., Dietrich K.A., Stephenson A.H., Ellsworth M.L., Sprague R.S. (2012). Prostacyclin receptor-mediated ATP release from erythrocytes requires the voltage-dependent anion channel. Am. J. Physiol. Heart Circ. Physiol..

[36] Sridharan M., Adderley S.P., Bowles E.A., Egan T., Stephenson A.H., Ellsworth M.L., Sprague R.S. (2010). Pannexin 1 is the conduit for low oxygen tension-induced ATP release from human erythrocytes. Am. J. Physiol. Heart Circ. Physiol..

[37] Shaskey D.J., Green G.A. (2000). Sports Haematology. Sports Med..

[38] Mao T.-Y., Fu L.-L., Wang J.-S. (2011). Hypoxic exercise training causes erythrocyte senescence and rheological dysfunction by depressed Gardos channel activity. J. Appl. Physiol..

[39] Mairbäurl H., Ruppe F.A., Bärtsch P. (2013). Role of Hemolysis in Red Cell Adenosine Triphosphate Release in Simulated Exercise Conditions In Vitro. Med. Sci. Sports Exerc..

[40] Sikora J., Orlov S.N., Furuya K., Grygorczyk R. (2014). Hemolysis is a primary ATP-release mechanism in human erythrocytes. Blood.

[41] Huisjes R., Bogdanova A., Van Solinge W., Schiffelers R., Kaestner L., Van Wijk R. (2018). Squeezing for Life—Properties of Red Blood Cell Deformability. Front. Physiol..

[42] Thomas S.L.Y. (2014). Intravascular hemolysis: The sacrifice of few…. Blood.

[43] Hahn A.W., Gill D.M., Pal S.K., Agarwal N. (2017). The future of immune checkpoint cancer therapy after PD-1 and CTLA-4. Immunotherapy.

[44] Kourie H.R., Awada G.G., Awada A. (2017). The second wave of immune checkpoint inhibitor tsunami: Advance, challenges and perspectives. Immunotherapy.

[45] Darvin P., Toor S.M., Nair V.S., Elkord E. (2018). Immune checkpoint inhibitors: Recent progress and potential biomarkers. Exp. Mol. Med..

[46] Di Virgilio F., Sarti A.C., Falzoni S., De Marchi E., Adinolfi E. (2018). Extracellular ATP and P2 purinergic signalling in the tumour microenvironment. Nat. Rev. Cancer.

[47] Allard D., Allard B., Stagg J. (2019). On the mechanism of anti-CD39 immune checkpoint therapy. J. Immunother. Cancer.

[48] Vultaggio-Poma V., Sarti A.C., Di Virgilio F. (2020). Extracellular ATP: A Feasible Target for Cancer Therapy. Cells.

[49] Campos-Contreras A.D.R., Díaz-Muñoz M., Vázquez-Cuevas F.G. (2020). Purinergic Signaling in the Hallmarks of Cancer. Cells.

[50] Allard B., Longhi M.S., Robson S.C., Stagg J. (2017). The ectonucleotidases CD39 and CD73: Novel checkpoint inhibitor targets. Immunol. Rev..

[51] Allard D., Chrobak P., Allard B., Messaoudi N., Stagg J. (2019). Targeting the CD73-adenosine axis in immuno-oncology. Immunol. Lett..

[52] Yu F.T.H., Chen X., Wang J., Qin B., Villanueva F.S. (2016). Low Intensity Ultrasound Mediated Liposomal Doxorubicin Delivery Using Polymer Microbubbles. Mol. Pharm..

[53] Liang X., Xu Y., Gao C., Zhou Y., Zhang N., Dai Z. (2018). Ultrasound contrast agent microbubbles with ultrahigh loading capacity of camptothecin and floxuridine for enhancing tumor accumulation and combined chemotherapeutic efficacy. NPG Asia Mater..

[54] Amate M., Goldgewicht J., Sellamuthu B., Stagg J., Yu F.T. (2020). The effect of ultrasound pulse length on microbubble cavitation induced antibody accumulation and distribution in a mouse model of breast cancer. Nanotheranostics.

[55] Liu H.-L., Hsieh H.-Y., Lu L.-A., Kang C.-W., Wu M.-F., Lin C.-Y. (2012). Low-pressure pulsed focused ultrasound with microbubbles promotes an anticancer immunological response. J. Transl. Med..

[56] Bulner S., Prodeus A., Gariepy J., Hynynen K., Goertz D.E. (2019). Enhancing Checkpoint Inhibitor Therapy with Ultrasound Stimulated Microbubbles. Ultrasound Med. Biol..

[57] Li N., Tang J., Yang J., Zhu B., Wang X., Luo Y., Yang H., Jang F., Zou J., Liu Z. (2021). Tumor perfusion enhancement by ultrasound stimulated microbubbles potentiates PD-L1 blockade of MC38 colon cancer in mice. Cancer Lett..

[58] Belcik J.T., Davidson B.P., Xie A., Wu M.D., Yadava M., Qi Y., Liang S., Chon C.R., Ammi A.Y., Field J. (2017). Augmentation of Muscle Blood Flow by Ultrasound Cavitation Is Mediated by ATP and Purinergic Signaling. Circulation.

[59] Hauben M., Hung E.Y., Hanretta K.C., Bangalore S., Snow V. (2015). Safety of Perflutren Ultrasound Contrast Agents: A Disproportionality Analysis of the US FAERS Database. Drug Saf..

[60] Tse W.-K., Leung P.T. (2006). Theory of light emission in sonoluminescence as thermal radiation. Phys. Rev. E.

[61] Mathias W., Tsutsui J.M., Tavares B.G., Fava A.M., Aguiar M.O., Borges B.C., Oliveira M.T., Soeiro A., Nicolau J., Ribeiro H.B. (2019). Sonothrombolysis in ST-Segment Elevation Myocardial Infarction Treated with Primary Percutaneous Coronary Intervention. J. Am. Coll. Cardiol..

[62] Carson A.R., McTiernan C.F., Lavery L., Grata M., Leng X., Wang J., Chen X., Villanueva F.S. (2012). Ultrasound-Targeted Microbubble Destruction to Deliver siRNA Cancer Therapy. Cancer Res..

[63] Chen S., Shimoda M., Wang M.-Y., Ding J., Noguchi H., Matsumoto S., Grayburn P.A. (2010). Regeneration of pancreatic islets in vivo by ultrasound-targeted gene therapy. Gene Therapy.

[64] Kotopoulis S., Dimcevski G., Gilja O.H., Hoem D., Postema M. (2013). Treatment of human pancreatic cancer using combined ultrasound, microbubbles, and gemcitabine: A clinical case study. Med Phys..

[65] Kopechek J.A., Carson A.R., McTiernan C.F., Chen X., Klein E.C., Villanueva F.S. (2016). Cardiac Gene Expression Knockdown Using Small Inhibitory RNA-Loaded Microbubbles and Ultrasound. PLoS ONE.

[66] Chen X., Wang J., Pacella J.J., Villanueva F.S. (2016). Dynamic Behavior of Microbubbles during Long Ultrasound Tone-Burst Excitation: Mechanistic Insights into Ultrasound-Microbubble Mediated Therapeutics Using High-Speed Imaging and Cavitation Detection. Ultrasound Med. Biol..

[67] Chen X., Leeman J.E., Wang J., Pacella J.J., Villanueva F.S. (2014). New Insights into Mechanisms of Sonothrombolysis Using Ultra-High-Speed Imaging. Ultrasound Med. Biol..

[68] Yu F.T., Chen X., Straub A.C., Pacella J.J. (2017). The Role of Nitric Oxide during Sonoreperfusion of Microvascular Obstruction. Theranostics.

[69] Abrahao A., Meng Y., Llinas M., Huang Y., Hamani C., Mainprize T., Aubert I., Heyn C., Black S.E., Hynynen K. (2019). First-in-human trial of blood–brain barrier opening in amyotrophic lateral sclerosis using MR-guided focused ultrasound. Nat. Commun..

[70] Deprez J., Lajoinie G., Engelen Y., De Smedt S., Lentacker I. (2021). Opening doors with ultrasound and microbubbles: Beating biological barriers to promote drug delivery. Adv. Drug Deliv. Rev..

[71] Kooiman K., Roovers S., Langeveld S.A., Kleven R.T., Dewitte H., O’Reilly M.A., Escoffre J.-M., Bouakaz A., Verweij M.D., Hynynen K. (2020). Ultrasound-Responsive Cavitation Nuclei for Therapy and Drug Delivery. Ultrasound Med. Biol..

[72] Fan Z., E Kumon R., Deng C.X. (2014). Mechanisms of microbubble-facilitated sonoporation for drug and gene delivery. Ther. Deliv..

[73] Helfield B., Chen X., Watkins S., Villanueva F.S. (2016). Biophysical insight into mechanisms of sonoporation. Proc. Natl. Acad. Sci. USA.

[74] Beekers I., Vegter M., Lattwein K.R., Mastik F., Beurskens R., Van Der Steen A.F., De Jong N., Verweij M.D., Kooiman K. (2020). Opening of endothelial cell–cell contacts due to sonoporation. J. Control. Release.

[75] Karshafian R., Bevan P.D., Williams R., Samac S., Burns P.N. (2009). Sonoporation by Ultrasound-Activated Microbubble Contrast Agents: Effect of Acoustic Exposure Parameters on Cell Membrane Permeability and Cell Viability. Ultrasound Med. Biol..

[76] Hu Y., Wan J.M., Yu A.C.H. (2013). Membrane Perforation and Recovery Dynamics in Microbubble-Mediated Sonoporation. Ultrasound Med. Biol..

[77] Kumon R., Aehle M., Sabens D., Parikh P., Han Y., Kourennyi D., Deng C. (2009). Spatiotemporal Effects of Sonoporation Measured by Real-Time Calcium Imaging. Ultrasound Med. Biol..

[78] van Rooij T., Skachkov I., Beekers I., Lattwein K.R., Voorneveld J.D., Kokhuis T.J., Bera D., Luan Y., van der Steen A.F., de Jong N. (2016). Viability of endothelial cells after ultrasound-mediated sonoporation: Influence of targeting, oscillation, and displacement of microbubbles. J. Control. Release.

[79] Haugse R., Langer A., Murvold E., Costea D., Gjertsen B., Gilja O., Kotopoulis S., De Garibay G.R., McCormack E. (2020). Low-Intensity Sonoporation-Induced Intracellular Signalling of Pancreatic Cancer Cells, Fibroblasts and Endothelial Cells. Pharmaceutics.

[80] Price R.J., Skyba D.M., Kaul S., Skalak T.C. (1998). Delivery of Colloidal Particles and Red Blood Cells to Tissue Through Microvessel Ruptures Created by Targeted Microbubble Destruction with Ultrasound. Circulation.

[81] Everbach E.C., Makin I.R., Azadniv M., Meltzer R.S. (1997). Correlation of ultrasound-induced hemolysis with cavitation detector output in vitro. Ultrasound Med. Biol..

[82] Sun T., Samiotaki G., Wang S., Acosta C., Chen C.C., Konofagou E.E. (2015). Acoustic cavitation-based monitoring of the reversibility and permeability of ultrasound-induced blood-brain barrier opening. Phys. Med. Biol..

[83] Fan Z., Liu H., Mayer M., Deng C.X. (2012). Spatiotemporally controlled single cell sonoporation. Proc. Natl. Acad. Sci. USA.

[84] Qin P., Xu L., Hu Y., Zhong W., Cai P., Du L., Jin L., Yu A.C. (2014). Sonoporation-Induced Depolarization of Plasma Membrane Potential: Analysis of Heterogeneous Impact. Ultrasound Med. Biol..

[85] Chen X., Leow R.S., Hu Y., Wan J.M.F., Yu A.C.H. (2014). Single-site sonoporation disrupts actin cytoskeleton organization. J. R. Soc. Interface.

[86] Leow R.S., Wan J.M.F., Yu A.C.H. (2015). Membrane blebbing as a recovery manoeuvre in site-specific sonoporation mediated by targeted microbubbles. J. R. Soc. Interface.

[87] Duan X., Zhou Q., Wan J.M.F., Yu A.C.H. (2021). Sonoporation generates downstream cellular impact after membrane resealing. Sci. Rep..

[88] Zeghimi A., Escoffre J.M., Bouakaz A. (2015). Role of endocytosis in sonoporation-mediated membrane permeabilization and uptake of small molecules: A electron microscopy study. Phys. Biol..

[89] Meijering B.D.M., Juffermans L.J.M., van Wamel A., Henning R., Zuhorn I., Emmer M., Versteilen A.M.G., Paulus W.J., van Gilst W., Kooiman K. (2009). Ultrasound and Microbubble-Targeted Delivery of Macromolecules Is Regulated by Induction of Endocytosis and Pore Formation. Circ. Res..

[90] Carson A.R., McTiernan C.F., Lavery L., Hodnick A., Grata M., Leng X., Wang J., Chen X., Modzelewski R.A., Villanueva F.S. (2011). Gene Therapy of Carcinoma Using Ultrasound-Targeted Microbubble Destruction. Ultrasound Med. Biol..

[91] Kovacs Z.I., Burks S.R., Frank J.A. (2018). Focused ultrasound with microbubbles induces sterile inflammatory response proportional to the blood brain barrier opening: Attention to experimental conditions. Theranostics.

[92] Belcik J.T., Mott B.H., Xie A., Zhao Y., Kim S., Lindner N.J., Ammi A., Linden J.M., Lindner J.R. (2015). Augmentation of Limb Perfusion and Reversal of Tissue Ischemia Produced by Ultrasound-Mediated Microbubble Cavitation. Circ. Cardiovasc. Imaging.

[93] Moccetti F., Belcik T., Latifi Y., Xie A., Ozawa K., Brown E., Davidson B.P., Packwood W., Ammi A., Huke S. (2020). Flow Augmentation in the Myocardium by Ultrasound Cavitation of Microbubbles: Role of Shear-Mediated Purinergic Signaling. J. Am. Soc. Echocardiogr..

